# Effects of a low dose of 3-nitrooxypropanol and a combination of direct-fed microbials on methane emission, oral swab bacterial community, and performance in dairy cows

**DOI:** 10.1186/s40104-026-01501-z

**Published:** 2026-10-07

**Authors:** W. Wang, S. Danesh Mesgaran, R. Gomez Exposito, A. Rytz, N. Walker, E. Soussan, S. Pruvost, D. R. Yáñez-Ruiz, P. Lund

**Affiliations:** 1 Department of Animal and Veterinary Sciences, AU Viborg – Research Centre, Aarhus University, Tjele, DK 8830 Denmark; 2 Nestlé Research, Animal Science, Nestlé Institute of Agricultural Sciences, Lausanne, 1000 Switzerland; 3 Agriculture and Food, Urrbrae, 5064 Australia; 4 DSM Nutritional Products, Animal Nutrition & Health, Basel, 4002 Switzerland; 5 Nestlé Research, Genomics and Automated Bioanalytics, Nestlé Institute of Food safety and Analytical Sciences, Lausanne, CH 1000 Switzerland; 6 Estación Experimental del Zaidín (CSIC), Profesor Albareda 1, Granada, 18008 Spain

**Keywords:** 3-Nitrooxypropanol, *Bacillus*, Direct-fed microbial, *Propionibacterium*

## Abstract

**Background:**

3-Nitrooxypropanol (3-NOP) can inhibit ruminal methane production by inactivating the key enzyme during methanogenesis. Combining 3-NOP and direct-fed microbials (DFM) can potentially provide a dual-action approach to reduce ruminal methane production. The objective of this study was to investigate the effects of supplementation of a low dose of 3-NOP and a mixture of DFM, on methane emissions, performance, and rumen microbial community of dairy cows.

**Results:**

Forty-eight Danish Holstein dairy cows were used in a 14-week randomized complete block design. Due to a mixing error, 3-NOP was included at an unexpected low dose (6.0 mg/kg DM). Cows were fed with one of four experimental diets: (1) control diet, and other treatment diets were control diet supplemented with (2) 3-NOP, or with (3) DFM (12 mg/kg *P. acidipropionici* strain P169 and 120 mg/kg *Bacillus subtilis* and *B. licheniformis* mixture), or with (4) a combination of 3-NOP and DFM. 3-NOP inclusion did not affect DMI, feed efficiency, or milk yield and composition. DFM supplementation reduced partial mixed ration intake and total DMI. 3-NOP supplementation significantly reduced CH_4_ production (g/d) (8%) and CH_4_ intensity (g/kg ECM) (9%) but did not affect CH_4_ yield (g/kg DMI). Furthermore, 3-NOP supplementation increased H_2_ production, yield, and intensity. DFM supplementation reduced milk yield from weeks 3 to 8 and ECM yield from weeks 3 to 7, but these effects diminished over time. 3-NOP alone or in combination with DFM resulted in higher abundance of *Megasphaera* and *Prevotella* genera in oral swab samples. DFM supplementation had no effect on gas emissions, and it only tended to improve feed efficiency (ECM/DMI, kg/kg) during the later stage of the experiment. DFM supplementation led to higher abundance of certain species within the Lactobacillaceae family particularly at later stages of the experiment.

**Conclusions:**

An unforeseen reduced dose of 3-NOP supplementation still can effectively mitigate methane emissions (both production and intensity) and increase H_2_ emissions without affecting DMI or performance. DFM supplementation reduced DMI and milk yield at the onset of the experiment, but the effects subsided over time, resulting in an increased feed efficiency by the end of the experiment.

**Supplementary Information:**

The online version contains supplementary material available at https://doi.org/10.1186/s40104-026-01501-z.

## Introduction

Enteric methane (CH_4_) emissions from livestock not only poses significant environmental challenges but also accounts for a loss of approximately 6% of ingested dietary gross energy in dairy cows [1]. The substantial methane mitigation effect of 3-nitrooxypropanol (3-NOP) in dairy cows has been extensively reported across multiple studies with dosages ranging between 40–200 mg/kg DM [2, 3]. This study aimed to evaluate a dose of 3-NOP of 60 mg/kg DM, but an unforeseen error led to an administered dose of only 6.0 mg/kg DM. However, evaluating reduced dosages of 3-NOP can still contribute to our understanding of its dose–response effects on methane emissions. To the best of our knowledge, no previous study has determined the effects of 3-NOP at doses below 30 mg/kg DM in dairy cows. An alternative methane mitigation approach is to explore other hydrogen sinks in the rumen. For example, propionate synthesis serves as a major alternative hydrogen sink that competes for hydrogen with methanogenesis. Enhancing propionate synthesis could, therefore, effectively reduce methane production [4]. Direct-fed microbials (DFM) have been widely studied for their potential to improve rumen fermentation, enhance feed digestion, and mitigate methane emissions [5]. Among DFM, *Propionibacterium acidipropionici* strain P169 has been shown to promote propionate synthesis in the rumen, thus potentially mitigating methane production [6]. Similarly, *Bacillus licheniformis* and *Bacillus subtilis* have demonstrated the ability to improve nutrient digestibility and utilization under both in vitro and in vivo conditions, benefiting beef and dairy cattle performance [7, 8]. Previous studies have attempted to combine the addition of 3-NOP with various strategies, such as the inclusion of fat, nitrate, and fumarate, and adjusting dietary concentrate level, to maximize methane mitigation and improve cow performance [9, 10]. However, to the best of our knowledge, no study has investigated the effects of the combination of 3-NOP and DFMs. Therefore, given their potentially complementary modes of action, we hypothesized that the combination of 3-NOP and DFM would have an additive effect on methane mitigation and cow performance, despite the lower-than-intended dose of 3-NOP used in the present study.

Global initiatives have been able to highlight the persistence of a core rumen microbiome across global geographical regions [11]. Studies have been able to unveil the association between rumen microbiome and certain animal phenotypes, including CH_4_ emissions [12]. As an alternative to the more invasive traditional methods for obtaining rumen contents for analysing the rumen microbiome, recent investigations have indicated that oral swabs could be a potentially reliable proxy for assessing the potential changes in rumen microbiome community [13, 14]. Nonetheless, sampling synchronization for oral swabbing is essential to ensure “rumen-dominant” swabs, as swab efficacy depends on the time elapsed in relation to animal’s rumination period [15, 16].

The aim of this study was to investigate the effects of 3-NOP supplementation and its interaction with a mixture of DFM with respect to methane emission, oral swab bacteria community composition, and performance in dairy cows. Additionally, given the unforeseen low dose of 3-NOP included in the diet, we also investigated the effect of reduced dose of 3-NOP on methane emissions to expand our understanding of its dose–response effects.

## Materials and methods

The experiment was conducted at Aarhus University, AU Viborg – Research Centre Foulum, Denmark, and animal experimental procedures and care of animal under study were carried out in accordance with the guidelines of the Danish Ministry of Food, Agriculture and Fisheries, The Danish Veterinary and Food Administration under act. No. 474 (May 15, 2014) and executive order No. 2028 (December 14, 2020) under consideration of the Arrive Guidelines [17]. The points included in the recently published paper about recommendations for testing methane mitigation feed additives were also considered [18].

### Animals and experimental design

The study was conducted with 48 lactating Danish Holstein dairy cows, including 24 primiparous and 24 multiparous cows. At the start of the experiment, the cows were on average (mean ± SD): 125 ± 34 and 112 ± 51 DIM; 624 ± 55.8, 686 ± 58.8 kg of BW; and 31.6 ± 3.83, 44.3 ± 6.95 kg/d of milk yield, for primiparous and multiparous cows, respectively. The cows were blocked into 12 blocks of four animals based on parity, DIM, and milk yield. Within each block, cows were randomly assigned to one of four diets in a randomized complete block design. The experiment consisted of a 2-week covariate period during which all cows were fed the experimental control (CON) diet, followed by a 12-week experimental period. Cows were housed in a loose-housing barn with concrete floors and cubicles fitted with rubber mattresses, and a mixture of straw and sawdust as bedding. Feed intake of individual cows was registered automatically using the Insentec RIC system (Insentec, Marknesse, the Netherlands), with feed bins calibrated every third week using a standard weight. Cows had free access to water. Two GreenFeed units (C-Lock Inc., South Dakota, USA) were placed at the end of the pen, positioned parallel to each other with a distance of 3 m. Each cow had access to both GreenFeed units throughout the entire experiment.

### Diets and feeding

The diets were formulated as partial mixed rations (PMR) according to the recommendations outlined in the NorFor system [19], based on an annual milk yield potential of 11,000 kg of ECM. The forage-to-concentrate ratio was 50:50 on DM basis for all diets. The four treatments were: (1) CON (basal PMR diet without any supplementation); (2) NOP (basal PMR diet with 3-NOP supplementation); (3) DFM (basal PMR diet with mixture of *Propionibacterium acidipropionici* strain P169 (2.0 × 10^11^ CFU/g), *Bacillus subtilis*, and *B. licheniformis* (3.2 × 10^9^ CFU/g) supplementation); and (4) NOPDFM (basal PMR diet with combination of 3-NOP and mixture of *P. acidipropionici* strain P169, *B. subtilis*, and *B. licheniformis* supplementation). The inclusion levels of *P. acidipropionici* strain P169, and the *B. subtilis* and *B. licheniformis* mixture were 12 and 120 mg/kg total DM, respectively. The 3-NOP source (3-nitrooxypropanol; Bovaer 10, DSM Nutritional Products, Switzerland) consisted of 10% active compound of 3-NOP and 90% of carrier substance (silicon dioxide + 1,2-propanediol) and was intended to be included at a concentration of 60 mg/kg DM, but due to an unforeseen error the actual observed concentration was only 6.0 mg/kg DM. All additives were mixed into the PMR. Forage constituted 50.1% of the total PMR DM and forage consisted of 21.5% first cut grass-clover silage, 24.6% first regrowth grass-clover silage, and 53.9% corn silage on DM basis. Concentrates constituted 49.9% of total PMR DM and was composed of spring barley, rapeseed meal, rapeseed cake, dried beet pulp. Ingredient proportion and chemical composition of the experimental diets are provided in Table 1, and chemical composition of ingredients is given in Table 2. The proportion of GreenFeed bait in the diet was based on the maximum allowable intake (approximately 1.35 kg/d) for each cow.

**Table 1:** Ingredients and chemical compositions of experimental basic diet (g/kg of DM unless otherwise stated, *n* = 2)^1^

Diets	Content
Ingredients	
Spring barley	108
Rapeseed meal	46.2
Rapeseed cake	174
Beet pulp (dried)	108
Corn silage	270
Grass-clover silage (first cut)	108
Grass-clover silage (first regrowth)	123
Sodium bicarbonate	9.61
Minerals^2^	5.75
ADE-Vitamin^3^	1.34
GreenFeed bait^4^	46.1
Chemical composition (mean ± SD)	
DM, g/kg of fresh matter	391 ± 10.5
Ash	67.5 ± 0.23
CP	174 ± 0.23
Crude fat	44.6 ± 0.49
NDF	316 ± 1.79
Starch	153 ± 2.24

^1^GreenFeed bait is not included in the calculation of the chemical composition of the diets as it was separately provided to the cows during the GreenFeed visit

^2^Concentration of minerals per kilogram of DM: 132 g of chloride, 65 g of magnesium, 50 g of phosphorus, 90 g of sodium, 1.5 g of potassium, 0.5 g of sulfur, 4,500 mg of zinc, 4,000 mg of manganese, 1,500 mg of copper, 225 mg of iodine, 50 mg of selenium, 25 mg of cobalt, 600,000 IU of vitamin A, 190,000 IU of vitamin D, 4,000 IU of vitamin E

^3^Concentration of vitamins per kilogram of DM: 10 mg of selenium, 5,000,000 IE of vitamin A, 200,000 IE of vitamin D, 10,000 IE of vitamin E

^4^GreenFeed bait (Primakalv Opdræt, DLG, Denmark) has a chemical composition of (g/kg of DM) CP, 180; fat, 40.0; ash, 66.0; Ca, 9.00; P, 4.10; Na, 2.00; Mg, 2.20; Zn, 0.075

**Table 2:** Chemical compositions of ingredients^1^ (mean ± SD; g/kg of DM unless otherwise noted, *n* = 2)

Item	Spring barley	Rapeseed cake	Rapeseed meal	Dried beet pulp	Corn silage	Grass-clover silage (first cut)	Grass-clover silage (first regrowth)	GreenFeed bait^2^
DM, g/kg of fresh matter	864 ± 2.05	875 ± 8.10	868 ± 6.35	907 ± 0.20	319 ± 8.15	174 ± 1.45	419 ± 5.25	876 ± 3.90
Ash	20.3 ± 0.13	72.2 ± 0.45	90.2 ± 4.03	77.7 ± 1.35	25.9 ± 0.97	93.9 ± 2.33	77.0 ± 0.18	73.8 ± 1.98
CP	119 ± 5.00	314 ± 6.25	370 ± 7.19	87.8 ± 0.94	73.1 ± 1.88	174 ± 0.31	151 ± 0.63	197 ± 0.31
Soluble CP^3^	253 ± 12.9	198 ± 2.94	206 ± 12.9	189 ± 5.57	721 ± 28.5	646 ± 13.2	568 ± 2.36	242 ± 2.80
Crude fat	28.5 ± 0.50	136 ± 11.0	45.0 ± 2.00	ND^4^	31.5 ± 0.50	34.0 ± 1.00	32.0 ± 0.00	46.5 ± 0.50
NDF	214 ± 25.0	285 ± 10.1	303 ± 7.20	354 ± 0.60	353 ± 4.55	377 ± 1.70	408 ± 4.40	290 ± 7.55
Starch	557 ± 9.05	2.50 ± 0.20	3.10 ± 0.20	ND	295 ± 12.6	ND	ND	138 ± 2.20

^1^Mean fermentation characteristics for first cut and first regrowth grass-clover silage and corn silage, respectively: pH = 3.80, 4.36, and 3.65; lactic acid = 73.4, 25.1, and 40.2 g/kg of DM; acetic acid = 23.8, 9.76, and 15.9 g/kg of DM; propionic acid = 0.14, 0.02, and 0.10 g/kg of DM; ethanol = 10.8, 4.36, and 6.08 g/kg of DM; ammonia N = 6.61%, 4.73%, and 7.91% of total N. No butyric acid was detected

^2^Ingredients of GreenFeed bait (Primakalv Opdræt, DLG, Denmark) are dried sugar beet pulp, 35.1%; distillers dried grains with solubles, 20.5%; wheat barn, 16.4%; rapeseed cake, 4.37%; rapeseed meal, 7.25%; malt sprouts, 5%; rye, 3.6%; barley, 2.8%; limestone, 1.27%; sunflower meal, 1.1%; sugar beet molasses, 0.88%; cane molasses, 0.7%; palm fat, 0.5%; salt (NaCl), 0.23%; vitamin micromineral premix, 0.3%

^3^Grams per kilograms of CP

^4^*ND* Not determined

Cows were fed one of the four PMRs ad libitum using automatic feed bins that recorded individual cow feed intake during each visit. Feed bins were filled three times daily (0700 h, 1330 h, and 1900 h) and emptied daily at 0700 h. Each cow had access only to bins allocated to the specific diet it was assigned to. Feed samples of PMR were collected directly from the mixer after mixing once per week for determining DM content and for subsequent nutrient analysis.

### Gas emissions

Gas emissions (CH_4_, CO_2_, and H_2_) from each cow were measured by GreenFeed system (C-Lock Inc., USA) throughout the entire experiment, which included a 2-week covariate period and 12-week measurement period. Details of the use of GreenFeed units are described by Maigaard et al. [10]. A commercial pelleted concentrate bait (Primakalv opdræt, DLG, Denmark, maximum 1,350 g/cow/d) was offered to cows during their visits to the GreenFeed units. The bait composition was as follows (DM basis): dried sugar beet pulp, 35.1%; DDGS, 20.5%; wheat bran, 16.4%; rapeseed cake, 4.37%; rapeseed meal, 7.25%; malt sprouts, 5.0%; rye, 3.6%; barley, 2.8%; limestone, 1.27%; sunflower meal, 1.1%; sugar beet molasses, 0.88%; cane molasses, 0.7%; palm fat, 0.5%; salt (NaCl), 0.23%; vitamin micromineral premix, 0.3%. During each visit, cows were allowed a maximum 6 drops (around 225 g) of concentrate, with a 40-s interval between drops. Cows were restricted to visit the GreenFeed units once every 4 h and no concentrate was dispensed if a cow visited the GreenFeed more frequently. Recovery test on CO_2_ (with an average recovery rate of 97.8% ± 2.22%) and calibrations were conducted every 3 weeks following the manufacturer’s instructions as described by Hristov et al. [20]. The daily average number of approved visits to GreenFeed units for each cow was 2.19 ± 1.14 (mean ± SD).

### Milk sampling and body weight

Cows were milked twice daily in a 2 × 12 side-by-side milking parlor (SAC, A/S S.A. Christensen & Co., Denmark) starting at 0700 h in the morning and 1630 h in the afternoon. Milk yield of each cow was recorded automatically during every milking. Milk samples from each cow were collected from the four consecutive milkings in each week from Tuesday afternoon to Thursday morning. Milk samples were stored at 5 °C with bronopol until milk composition analysis. Cows were weighed after each milking when exiting the milking parlor.

### Chemical analysis

The DM content was determined in feed ingredients and the PMR using a forced-air oven at 60 °C for 48 h, and dried samples were milled through a 1-mm screen before chemical analysis. Ash content was determined by combustion at 525 °C for 6 h. The nitrogen content of feed was analyzed by the Dumas method [21] using a Vario MAX CN (Elementar Analysesysteme GmbH, Germany) and CP content was calculated as 6.25 × total N. Crude fat was measured using Soxhlet extraction with petroleum ether (Soxtec 2050, Foss Analytical) after hydrolysis with HCl [22]. Starch content in feed was measured enzymatically [23]. Neutral detergent fibre (NDF) content was determined using a Fibertec M6 System (Foss Analytical, Denmark) using heat-stable α-amylase and sodium sulphite [24], and reported as ash-free NDF.

Milk crude protein, fat, lactose monohydrate, urea concentration were analyzed using a Milkoscan 7RM FT + (Foss Analytical, Denmark) at Eurofins Steins Laboratorium (Vejen, Denmark).

### Oral sample collection and metagenomics data analyses

Sample collection was carried out in week 2 of the covariate period, followed by weeks 6 and 12 of the measurement period. Staff were trained before sampling and oral samples were obtained roughly 4 h after the morning feeding using a buccal swab (Puritan HydraFlock, Puritan Diagnostics, USA) inserted laterally through the mouth of the cow coinciding with a regurgitation of a rumen bolus during rumination. Buccal swabs were then stored in vials and kept at −20 °C for further analysis.

Total DNA was extracted from 200 µL of saliva resuspended in DNA/RNA shield buffer (1:1, v/v; Cat. No. R1100-50, Zymo Research, Irvine, CA, USA) using ZymoBIOMICS 96 Magbead DNA Kit (Zymo Research, USA) by following the manufacturer's instructions. Eluted DNA quantification was performed with the Quant-iT Picogreen dsDNA Assay (Invitrogen, USA) and its size checked on the TapeStation (Agilent, USA) with Genomic DNA ScreenTape Analysis. Library preparation was performed with 100 ng of gDNA input according to the Illumina DNA Prep Reference Guide (Part # 1000000025416 v09 June 2020), and quality checks were done using Qubit before pooling the libraries in equimolarity. PE150 run was performed on NextSeq2000 with P3 reagent, loaded at 650 pM and with 2% PhiX ensuring optimal flow cell loading and high-quality metrics (QC30). Sequencing data were demultiplexed on the NextSeq with DRAGEN and confirming the equimolarity of pooling.

KneadData [25], as a wrapper script for quality control, was used for quality trimming and filtering, removal of adapter sequences, and discarding human contaminated reads. Bowtie2 [26] was utilized for host genome decontamination. The Kraken2-bracken system for the assignment of sequence reads to taxonomic labels as well as estimating the taxa abundance [27, 28]. Custom reference database encompassing the reference sequences from bacteria and the human genome was obtained to perform the taxonomic assignment.

### Calculations and statistical analysis

Data on individual dry matter intake (DMI) was calculated by summing the PMR intake and the intake of concentrate bait consumed during GreenFeed visits. Milk yield and the yield and concentration of fat, protein, and lactose, and milk urea concentrations, were calculated as weighted average from the four milkings in each week. Energy-corrected milk (ECM**)** yield (3.14 MJ/kg) was calculated using the formula described by Sjaunja et al. [29]: ECM (kg/d) = milk yield (kg/d) × (38.3 × milk fat (g/kg) + 24.2 × milk protein (g/kg) + 15.71 × milk lactose (g/kg) + 20.7)/3,140, where lactose refers to lactose monohydrate. To improve statistical robustness of the gas emission data from more GreenFeed visits, the gas data collected were averaged using rolling three-week intervals and used for subsequent statistical analysis. The averages of the data from weeks 1, 2, and 3 were presented as week 3, and so on, thus no data was presented as week 1 and 2 in the experimental period. For data regarding intake, feed efficiency, and gas yields (CO_2_, CH_4_, and H_2_), 17 observations from the CON group and 6 observations from the NOP group were omitted due to peculiar feed intake records; additionally, 1 observation from the NOPDFM group was excluded due to cow illness. Consequently, there were 120, 116, 118, and 106 observations from gas emission data included in the statistical analysis.

Data were analyzed in R (version 4.4.1) using linear mixed-effects models (LMM) with the ‘lme’ function from the ‘nlme’ package [30]. The rolling means were calculated and analyzed in the following model separately:

$$\begin{aligned} Y_{ijkl} &= \mu + NOP_{i} + DFM_{j} + Week_{k}\\ &\quad + (NOP \times DFM)_{ij} + (NOP \times Week)_{ik}\\ &\quad+ (DFM \times Week)_{jk} + (NOP \times DFM\\ &\quad\times Week)_{ijk}+ Block_{l} + Cov_{ijl} + E_{ijkl} \end{aligned}$$

Where *Y*_*ijkl*_ = dependent variable; *μ* = overall mean; *NOP*_*i*_ = fixed effect of 3-NOP supplementation; *DFM*_*j*_ = fixed effect of DFM supplementation; Week = fixed effect of experimental week (3 to 12, for instance, week1 = week 1 + 2 + 3; week2 = week 2 + 3 + 4 for DMI, milk yield and composition, and gas emission data); (*NOP* × *DFM*)_*ij*_ = interaction between 3-NOP and DFM supplementation; (*NOP* × *Week*)_*ik*_ = interaction between 3-NOP supplementation and experimental week; (*DFM* × *Week*)_*jk*_ = interaction between DFM supplementation and experimental week; (*NOP* × *DFM* × *Week*)_*ijk*_ = interaction between 3-NOP, DFM, and experimental week; *Block*_*l*_ = random effect of Block (1 to 12); *Cov*_*ijl*_ = effect of covariate; *E*_*ijkl*_ = random error assumed to be independent and normally distributed. The model included an autoregressive covariance structure (corAR1) as a repeated measures from the same cows across different experimental weeks that were included in the dataset. Like the previous model, cows were blocked based on parity; therefore, parity was not included as a fixed effect in the model. Least squares of means were presented in tables, and significance or trends were declared at *P* ≤ 0.05 and 0.05 < *P* ≤ 0.10, respectively.

Oral samples from the control and treatment groups were compared by diversity and differential abundance analyses. One sample from the control group was excluded from downstream analysis due to an insufficient number of reads. Permutational Multivariate Analysis of Variance and Wilcoxon test was used for computing the statistical significance for beta and alpha diversity, respectively. The R package ‘ALDEx2’ was used for detecting the within-group and between-group differential abundance analysis. Wilcoxon test was carried out for statistical analysis of the absolute-change analysis and Cohen’s d for effect-size estimation.

## Results

### Dry matter intake, milk yield and composition, and feed efficiency

3-NOP supplementation had no effect on total DMI or average daily BW gain (ADG) (*P* > 0.05; Table 3). In contrast, DFM supplementation significantly reduced both PMR and total DMI (on average by 0.8 and 0.75 kg, respectively, *P* < 0.05). 3-NOP supplementation did not affect the yields of milk, ECM, fat, protein, and lactose (*P* > 0.05; Table 4). Though DFM supplementation overall tended to decrease milk yield and the reduction effect of DFM supplementation on milk yield weakened over time (*P*_DFM × Week_ < 0.01). Specifically, DFM supplementation reduced milk yield from weeks 3 to 8 (*P* ≤ 0.01; Table S1), but no significant effects were observed in weeks 9 to 12 (*P* > 0.05). Similarly, ECM yield was reduced by DFM supplementation from weeks 3 to 7 (*P* < 0.05) and tended to be reduced at week 8 (*P* = 0.10).

**Table 3:** Effects of 3-NOP and direct-fed microbials (DFM) on feed intake, feed efficiency, and average daily gain^1^

Items^2^	Treatment^3^				SEM^4^	*P*-value^5^					
	CON	NOP	DFM	NOPDFM		NOP	DFM	Week	NOP × DFM	NOP × Week	DFM × Week
PMR DMI, kg/d	25.5	25.2	24.7	24.4	0.64	0.37	0.03	< 0.001	0.93	0.56	0.48
GreenFeed bait DMI, kg/d	0.34	0.26	0.31	0.30	0.03	0.14	0.86	< 0.001	0.26	0.89	0.18
Total DMI, kg/d	25.8	25.5	25.1	24.7	0.65	0.29	0.03	< 0.001	0.97	0.57	0.43
ADG, kg/d	0.67	0.62	0.59	0.53	0.08	0.42	0.25	−	0.97	−	−

^1^There were 17 observations from CON group and 6 observations from NOP group were omitted due to peculiar feed intake records, 1 observation from NOPDFM was omitted due to illness in a cow, and there were 12 observations in each group for ADG

^2^*PMR* Partially mixed ration, *DMI* Dry matter intake, *ADG* Average daily gain

^3^CON: without any supplementation; NOP; 3-NOP supplementation; DFM: mixture of *Propionibacterium acidipropionici* strain P169, *Bacillus subtilis*, and *B. licheniformis* supplementation; NOPDFM: combination of 3-NOP and mixture of *Propionibacterium acidipropionici* strain P169, *B. subtilis*, and *B. licheniformis* supplementation

^4^Largest values were reported, due to missing observations

^5^Due to the space limitation, the insignificant three-way interaction was not presented in the table. In all models, the average of the variable in the covariate period was included and always had a significance of *P* < 0.001

**Table 4:** Effects of 3-NOP and direct-fed microbials (DFM) on milk yield and composition^1^

Items	Treatment^3^				SEM^4^	*P*-value^5^					
	CON	NOP	DFM	NOPDFM		NOP	DFM	Week	NOP × DFM	NOP × Week	DFM × Week
Yield, kg/d											
Milk	34.0	35.6	35.5	34.1	1.45	0.99	0.06	< 0.001	0.90	0.30	< 0.001
ECM^2^	35.7	36.4	35.9	35.9	1.15	0.57	0.74	0.04	0.63	0.88	0.07
Fat	1.38	1.38	1.39	1.40	0.04	0.82	0.63	0.81	0.91	0.90	0.03
Protein	1.34	1.39	1.34	1.34	0.05	0.40	0.35	< 0.01	0.31	0.89	0.34
Lactose	1.68	1.73	1.67	1.67	0.07	0.50	0.26	< 0.001	0.54	0.66	0.25
Composition,%											
Fat	4.16	3.98	4.07	4.16	0.09	0.54	0.52	< 0.001	0.07	0.83	0.23
Protein	3.93	3.95	3.93	3.92	0.03	0.84	0.65	< 0.001	0.53	0.18	0.39
Lactose	4.88	4.88	4.88	4.86	0.02	0.54	0.44	0.25	0.62	0.84	0.38
Urea, mmol/L	3.89	3.91	3.78	3.66	0.08	0.50	0.01	< 0.01	0.31	0.74	0.67
ECM/DMI, kg/kg	1.42	1.43	1.43	1.46	0.03	0.48	0.38	< 0.001	0.77	0.51	< 0.01

^1^There were 479 observations in total, 1 observation from NOPDFM was omitted due to illness in a cow. For ECM/DMI there were 17 extra observations from CON group and 6 extra observations from NOP group were omitted due to peculiar feed intake records

^2^ECM yield (Sjaunja et al. [29])

^3^CON: without any supplementation; NOP: 3-NOP supplementation; DFM: mixture of *Propionibacterium acidipropionici* strain P169, *Bacillus subtilis*, and *B. licheniformis* supplementation; NOPDFM: combination of 3-NOP and mixture of *Propionibacterium acidipropionici* strain P169, *B. subtilis*, and *B. licheniformis* supplementation

^4^Largest values were reported, due to missing observations

^5^Due to the space limitation, the insignificant three-way interaction was not presented in the table. In all models, the average of the variable in the covariate period was included and always had a significance of *P* < 0.001

**Fig. 1:**
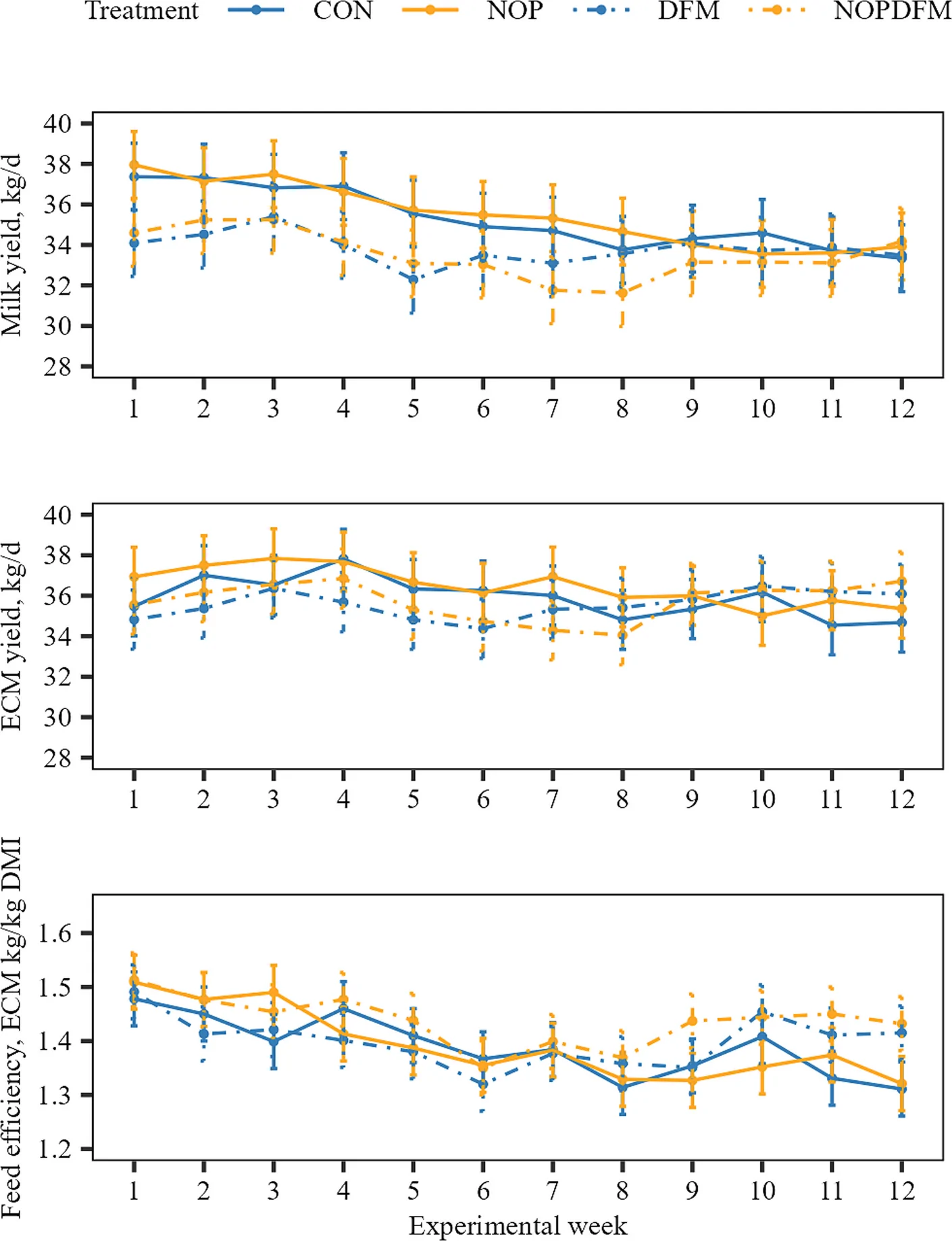
Effects of 3-NOP and direct-fed microbials (DFM) on milk yield, ECM yield, and feed efficiency over time. CON: without any supplementation; NOP: 3-NOP supplementation; DFM: mixture of *Propionibacterium acidipropionici* strain P169, *Bacillus subtilis*, and *B. licheniformis* supplementation; NOPDFM: combination of 3-NOP and mixture of *Propionibacterium acidipropionici* strain P169, *B. subtilis*, and *B. licheniformis* supplementation

A significant interaction between DFM supplementation and experimental week was observed for feed efficiency (*P*_DFM × week_ = 0.046). DFM supplementation had no effect on feed efficiency from weeks 3 to 11 (*P* > 0.05; Table S2), but tended to increase feed efficiency in week 12 (*P* = 0.08; Fig. 1). Overall, 3-NOP supplementation had no effect on feed efficiency (ECM/DMI) (*P* > 0.05; Table 4).

### Gas emissions

No interaction between 3-NOP and DFM supplementation was observed for gas emissions (*P* > 0.05; Table 5). The effect of DFM supplementation on CO_2_ intensity changed over time (*P*_DFM × Week_ = 0.01), as DFM supplementation increased CO_2_ intensity by 2% in week 3, with the increase progressively diminished and reduced CO_2_ intensity by 6% in week 12 (Table S3). Neither 3-NOP nor DFM supplementation significantly affected CO_2_ production, yield, or intensity (*P* > 0.05). CH_4_ production and intensity were reduced by 3-NOP supplementation (respectively 8% and 9%, *P* < 0.05), while CH_4_ yield remained unaffected (*P* > 0.05). DFM supplementation had no effect on CH_4_ emissions (production, yield, and intensity) (*P* > 0.05). H_2_ production, yield, and intensity were significantly increased by 3-NOP supplementation (64%, 59%, and 50%, respectively, *P* < 0.001), but were not affected by DFM inclusion (*P* > 0.05).

**Table 5:** Effects of 3-NOP and direct-fed microbials (DFM) on gas emission^1^

Items	Treatment^2^				SEM^3^	*P*-value^4^					
	CON	NOP	DFM	NOPDFM		NOP	DFM	Week	NOP × DFM	NOP × Week	DFM × Week
CO_2_, g/d	13,846	13,583	13,725	13,624	373	0.49	0.88	< 0.001	0.76	0.53	0.66
CO_2_ yield, g/kg DMI	542	538	542	559	13.0	0.55	0.33	0.41	0.35	0.49	0.90
CO_2_ intensity, g/kg ECM	405	380	379	388	12.5	0.53	0.46	< 0.001	0.15	0.40	0.01
CH_4_, g/d	374	334	359	341	12.0	< 0.01	0.71	< 0.01	0.30	0.55	0.90
CH_4_ yield, g/kg DMI	14.5	13.3	14.1	14.1	0.46	0.15	0.65	0.45	0.14	0.31	0.51
CH_4_ intensity, g/kg ECM	10.9	9.28	9.94	9.73	0.39	0.02	0.48	< 0.001	0.07	0.57	0.13
H_2_, g/d	2.73	4.17	2.68	4.71	0.28	< 0.001	0.30	0.06	0.20	0.93	0.71
H_2_ yield, g/kg DMI	0.11	0.16	0.11	0.19	0.01	< 0.001	0.09	0.27	0.11	0.97	0.50
H_2_ intensity, g/kg ECM	0.08	0.11	0.08	0.13	0.01	< 0.001	0.29	< 0.01	0.10	0.70	0.67

^1^There were 460 observations in total, 120, 116, 118, and 106 observations for CON, NOP, DFM, and NOPDFM group, respectively. In addition, for CO_2_ yield, CH_4_ yield, and H_2_ yield, there were 17 extra observations from CON group and 6 extra observations from NOP group were omitted due to peculiar feed intake records

^2^CON: without any supplementation; NOP: 3-NOP supplementation; DFM: mixture of *Propionibacterium acidipropionici* strain P169, *Bacillus subtilis*, and *B. licheniformis* supplementation; NOPDFM: combination of 3-NOP and mixture of *Propionibacterium acidipropionici* strain P169, *B. subtilis*, and *B. licheniformis* supplementation

^3^Largest values were reported, due to missing observations

^4^Due to the space limitation, the insignificant three-way interaction was not presented in the table. In all models, the average of the variable in the covariate period was included and always had a significance of *P* < 0.001

On a weekly basis, a negative interaction between 3-NOP and DFM supplementation was observed for CH_4_ intensity from weeks 3 to 10 (*P*_NOP × DFM_ < 0.05; Table S4). The combination of 3-NOP and DFM supplementation led to a smaller reduction in CH_4_ intensity compared to 3-NOP alone. 3-NOP supplementation significantly reduced CH_4_ production at weeks 3, 4, 5, 6, and 11 (*P* < 0.05; Table S4) and showed a tendency to reduce CH_4_ production at weeks 7 and 10 (both *P* = 0.08). In addition, a similar negative interaction between 3-NOP and DFM supplementation was observed for CH_4_ yield in weeks 4, 5, 8, 9, and 10 (*P* ≤ 0.04; Table S4) and the interaction tended to be significant in weeks 3 and 6 (*P* < 0.10). In contrary to CH_4_ yield and intensity, positive interactions between 3-NOP and DFM supplementation for H_2_ yield (weeks 5 and 7) and intensity (weeks 4 and 5) were noted (*P* < 0.05; Table S5), with the combination of 3-NOP and DFM supplementation resulting in a greater increase in H_2_ yield and intensity than either supplement alone. Furthermore, DFM supplementation increased H_2_ yield in week 4 and increased H_2_ intensity in week 6 (*P* < 0.05).

### Shotgun metagenomics data analyses

A total of 143 oral samples were processed for shotgun metagenomic analysis. With the extraction method, good quality gDNA was obtained for all samples despite low yield (~0.5 ng/µL). For the library preparation, the maximum quantity of gDNA possible was used to generate the libraries for sequencing. Sequencing data were demultiplexed with DRAGEN, and samples were sorted by their attributed indexes. The number of reads per sample was homogeneous, with an average sequencing depth of approximately 7 Gb per sample. On the dataset five samples yielded lower sequencing output (< 1 Gb), including one of which was excluded due to an insufficient number of reads (< 0.5 M PE Read). All samples (except one) were retained in the dataset and included in downstream analyses where appropriate, with sequencing depth taken into account during data normalization and interpretation. After first cleaning, the average raw and classified reads were 25.37 ± 5.18 (mean ± SD), and 8.86 ± 4.92 million reads per sample. On average 10.9% of reads were dropped during quality filtering step and around 40.4% on average reads were dropped during host decontamination step.

### The structure and differential abundance of the bacterial community

**Fig. 2:**
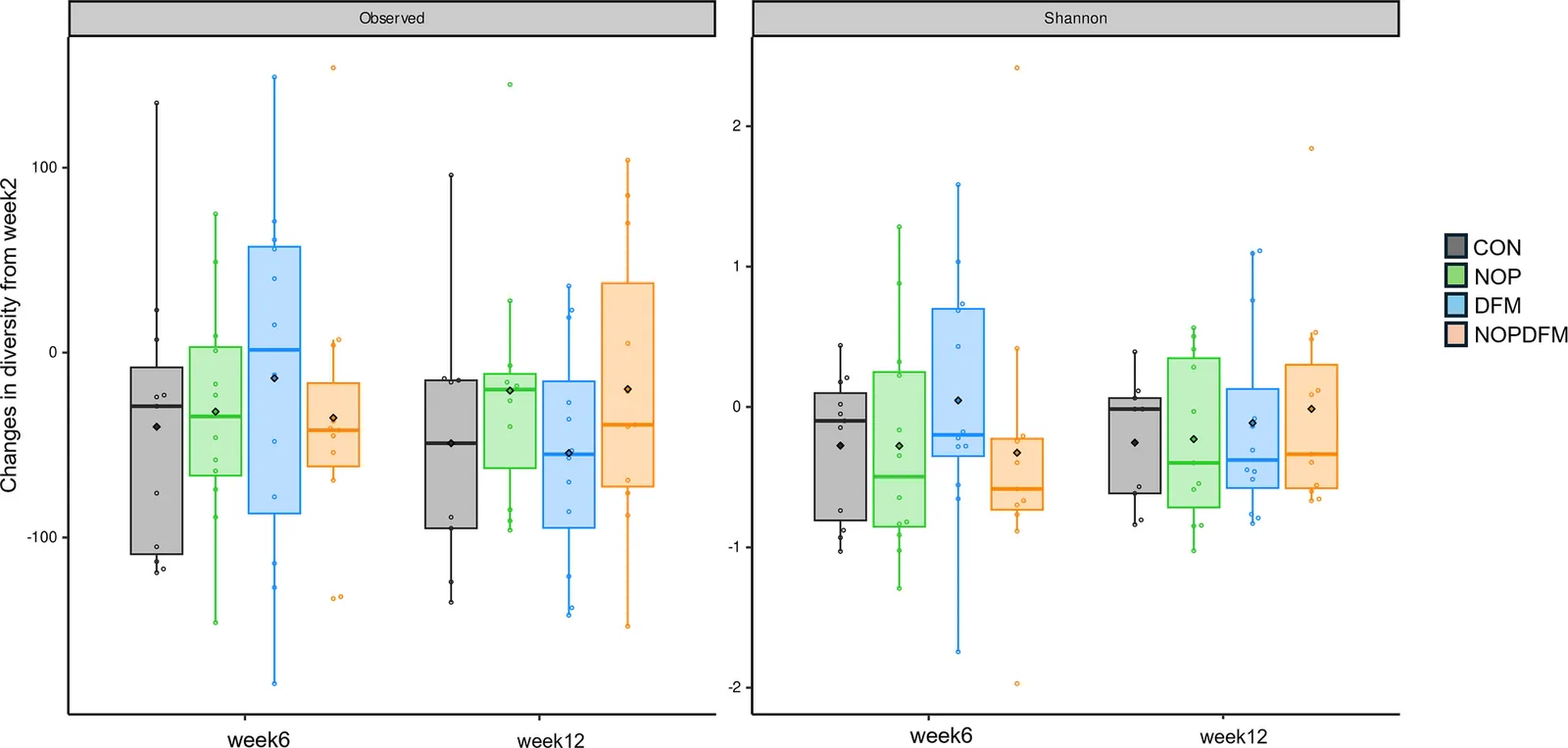
Effects of 3-NOP and direct-fed microbials (DFM) on alpha diversity depicting changes of oral swab microbiome diversity at weeks 6 and 12 of the experiment period in relation to week 2 covariate period between treatment groups for Observed and Shannon index. CON: without any supplementation; NOP: 3-NOP supplementation; DFM: mixture of *Propionibacterium acidipropionici* strain P169, *Bacillus subtilis*, and *B. licheniformis* supplementation; NOPDFM: combination of 3-NOP and mixture of *Propionibacterium acidipropionici* strain P169, *B. subtilis*, and *B. licheniformis* supplementation

**Fig. 3:**
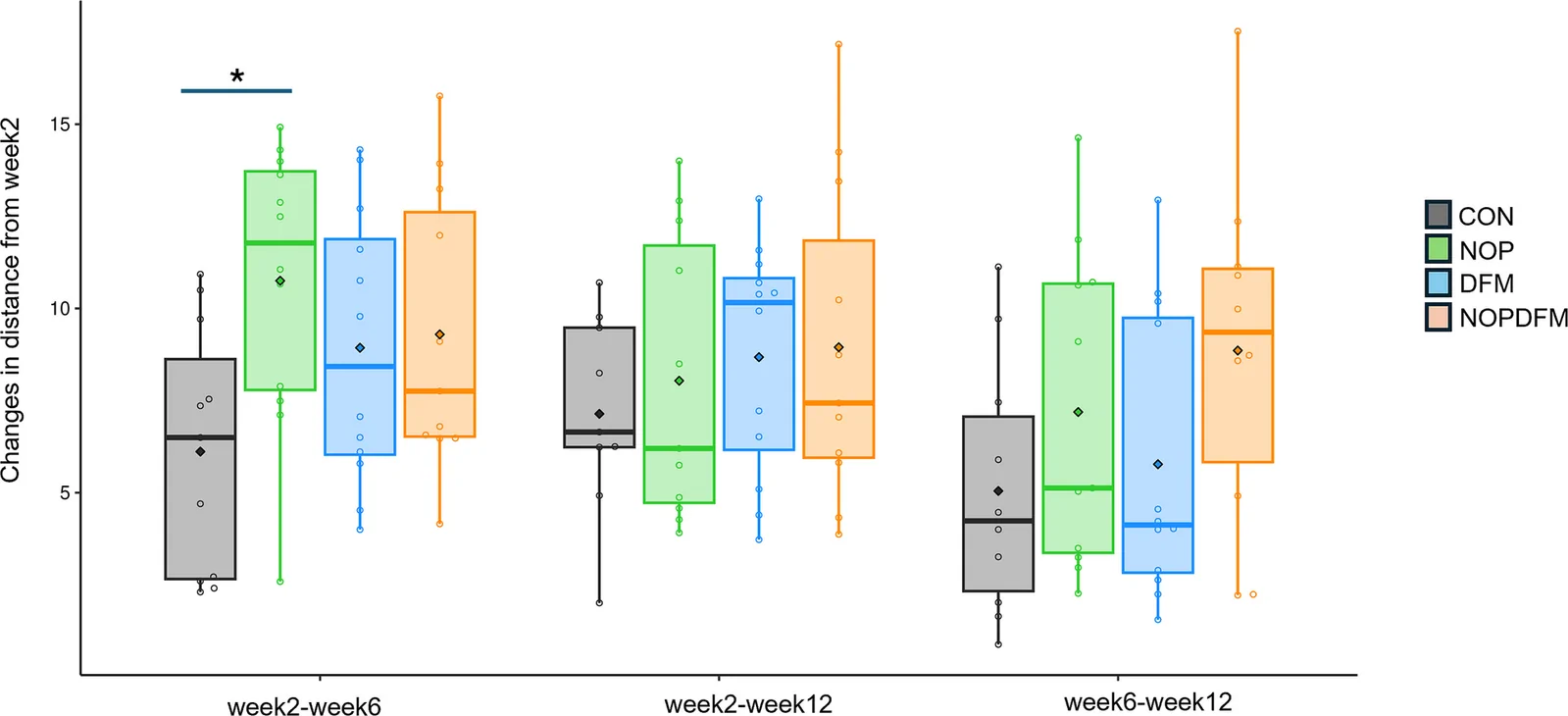
Effects of 3-NOP and direct-fed microbials (DFM) on beta diversity of oral swab microbiome at weeks 6 and 12 of the experiment period in relation to week 2 covariate period. For each animal, the change in distance at weeks 6 and 12 relative to week 2 of covariate period was calculated, and the results were compared across treatment groups. ^*^P < 0.05. CON: without any supplementation; NOP: 3-NOP supplementation; DFM: mixture of *Propionibacterium acidipropionici* strain P169, *Bacillus subtilis*, and *B. licheniformis* supplementation; NOPDFM: combination of 3-NOP and mixture of *Propionibacterium acidipropionici* strain P169, *B. subtilis*, and *B. licheniformis* supplementation

**Fig. 4:**
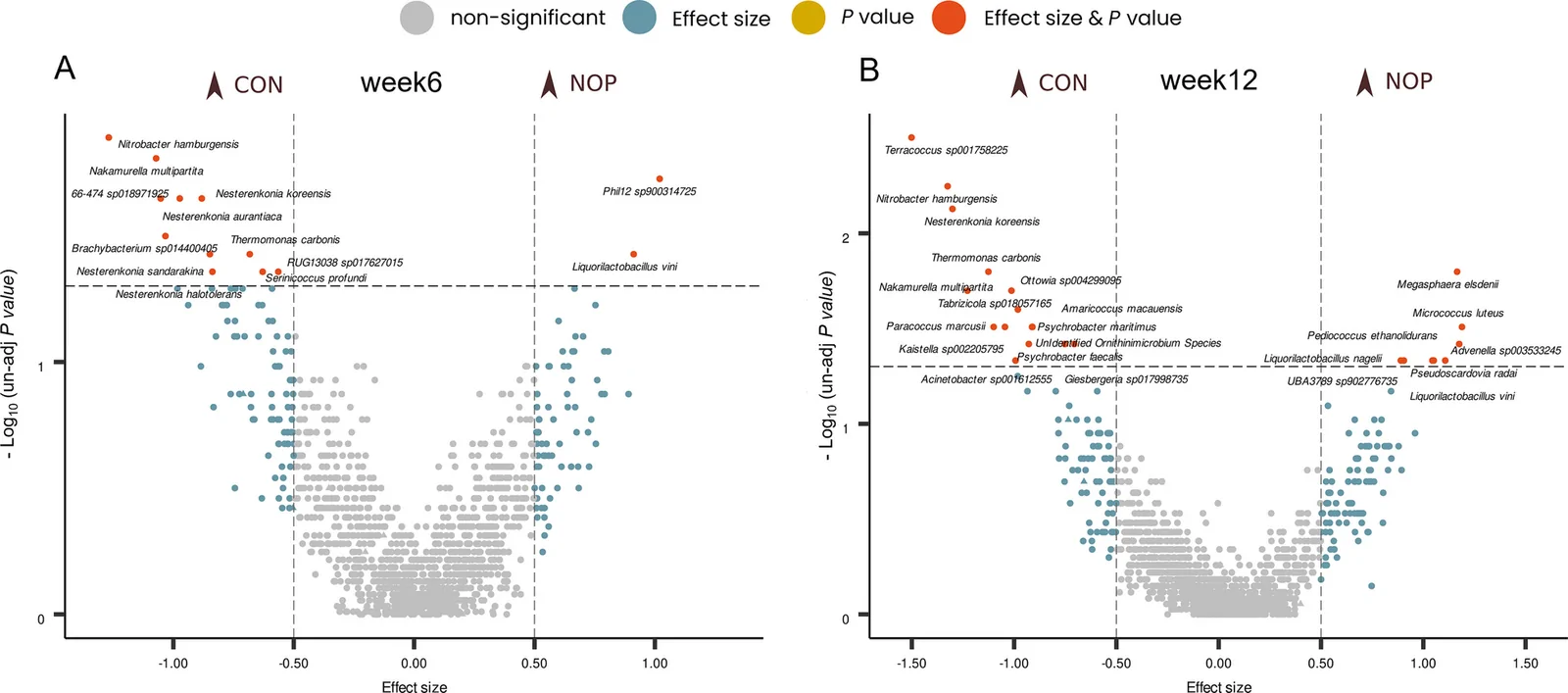
Effects of 3-NOP and direct-fed microbials (DFM) on differential abundance analysis of oral swab microbiome at weeks 6 and 12 of the experiment period in relation to week 2 covariate period. **A** and **B** Differential abundance changes between CON and NOP at weeks 6 and 12 of the experimental period. For each animal, absolute changes were computed at weeks 6 and 12 relative to week 2 of covariate period. Wilcoxon test and Cohen’s d were utilized for statistical comparison and effect size measurement, respectively. Green dots depict bacterial species that only passed the effect size threshold. Red dots depict bacterial species that passed both effect size and *P*-value thresholds. CON: Without any supplementation; NOP: 3-NOP supplementation

**Fig. 5:**
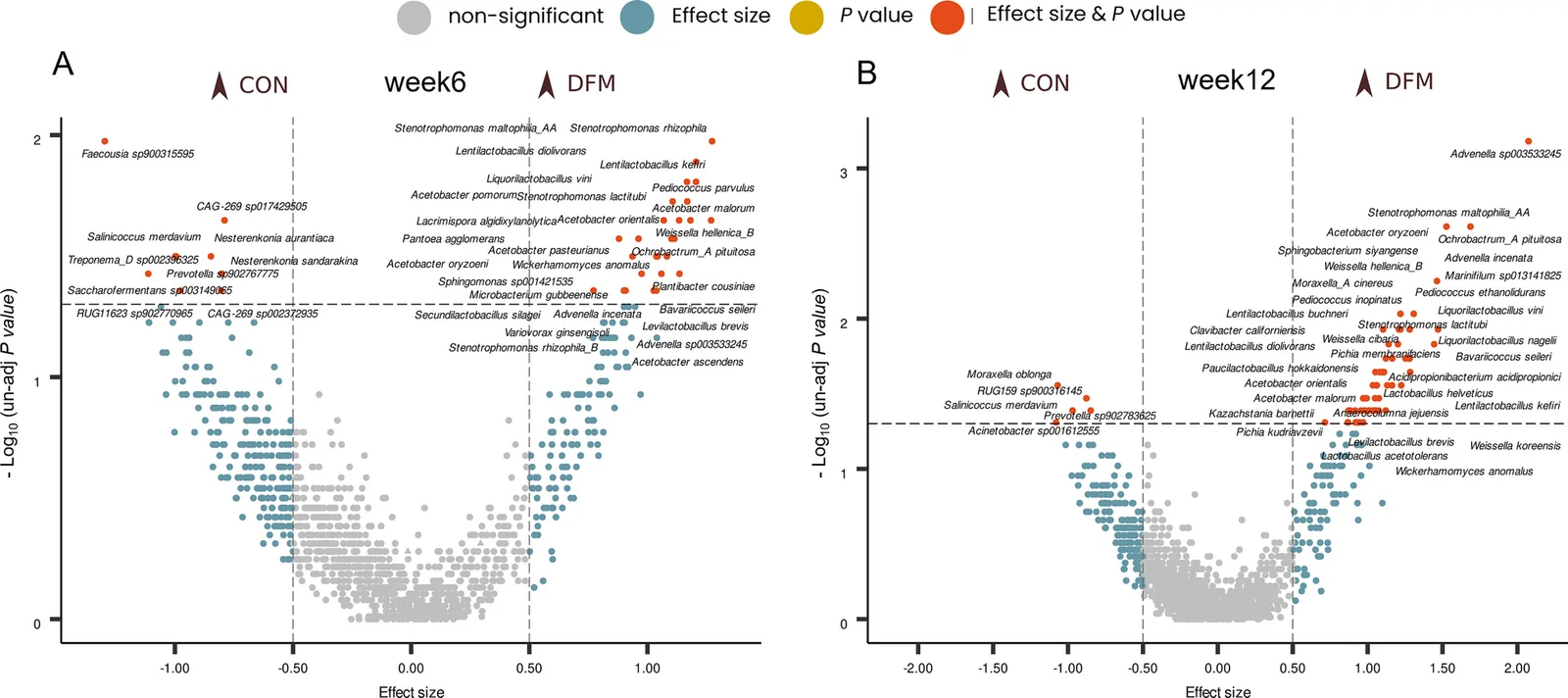
Effects of 3-NOP and direct-fed microbials (DFM) on differential abundance analysis of oral swab microbiome at weeks 6 and 12 of the experiment period in relation to week 2 covariate period. **A** and **B** Differential abundance changes between CON and DFM at weeks 6 and 12 of the experimental period. For each animal, absolute changes were computed at weeks 6 and 12 relative to week 2 of covariate period. Wilcoxon test and Cohen’s d were utilized for statistical comparison and effect size measurement, respectively. Green dots depict bacterial species that only passed the effect size threshold. Red dots depict bacterial species that passed both effect size and *P-*value thresholds. CON: without any supplementation; DFM: mixture of *Propionibacterium acidipropionici* strain P169, *Bacillus subtilis*, and *B. licheniformis* supplementation

**Fig. 6:**
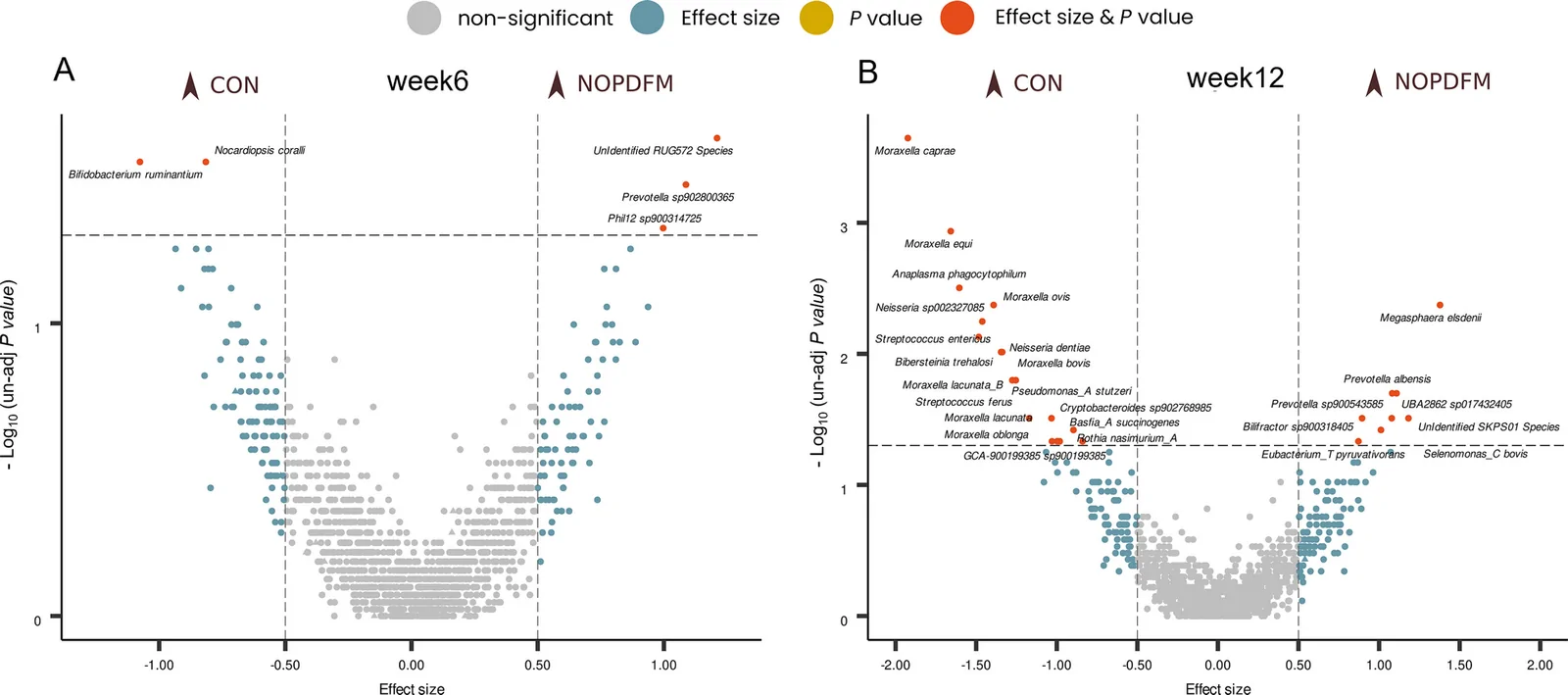
Effects of 3-NOP and direct-fed microbials (DFM) on differential abundance analysis of oral swab microbiome at weeks 6 and 12 of the experiment period in relation to week 2 covariate period. **A** and **B** Differential abundance changes between CON and NOPDFM at weeks 6 and 12 of the experimental period. For each animal, absolute changes were computed at weeks 6 and 12 relative to week 2 of covariate period. Wilcoxon test and Cohen’s d were utilized for statistical comparison and effect size measurement, respectively. Green dots depict bacterial species that only passed the effect size threshold. Red dots depict bacterial species that passed both effect size and *P-*value thresholds. CON: without any supplementation; NOPDFM: combination of 3-NOP and mixture of *Propionibacterium acidipropionici* strain P169, *B. subtilis*, and *B. licheniformis* supplementation

Alpha diversity analyses using both Shannon and Observed indexes, showed that the differences in bacterial diversity between groups were not statistically significant (Fig. 2). Beta diversity analysis showed an increased Aitchison distance for the 3-NOP treatment at week 6 of the measurement period in comparison with CON (Fig. 3). Moreover, at week 12 of the measurement period there was a trend for increased distance change in the NOPDFM treatment in comparison with CON (Fig. 3). The two most abundant phyla were Firmicutes and Bacteroidota, which altogether accounted for 61.8% of the total reads. The addition of 3-NOP and DFM resulted in considerable changes in the bacterial community (Figs. 4, 5, 6). The changes in abundance for each animal were computed at weeks 6 and 12 of the measurement period and related to data from week 2 of the covariate period. Supplementation of 3-NOP resulted in higher abundance of the *Megasphaera* genus at week 12 compared to CON (*P* < 0.05). The abundance of *Megasphaera elsdenii* was higher upon 3-NOP supplementation than CON (*P* < 0.05). Supplementation of the DFM led to higher abundance of both *Lentilactobacillus* and *Lactobacillus* genera in weeks 6 and 12 of the measurement period (*P* < 0.05). The combination of 3-NOP and DFM in the diet of animals resulted in higher abundance of *Megasphaera elsdenii*, *Prevotella albensis,* and *Prevotella* sp. 900543585 compared to CON (*P* < 0.05). Moreover, *Selenomonas bovis* abundance in week 12 of the measurement period tended to be higher in the NOPDFM group in comparison with CON.

### Metagenomic functional profiling and metabolic pathway reorganization

To evaluate the functional potential of the rumen microbiome, metagenomic data was mapped to KEGG modules and visualized via volcano plots (Fig. S1). At week 12, the Pentose Phosphate Pathway (M00004) was significantly enriched in the 3NOP group compared to the Control. Conversely, core structural pathways for methanogenesis (M00356, M00357) remained stable at the baseline centre line, depicting no marked differences between the two experimental groups.

At week 6 two specific functional modules achieved evident enrichment in the DFM group, i.e., Biotin biosynthesis via pimeloyl-ACP/CoA (M00123) and Biotin biosynthesis via the BioW pathway (M00577; Fig. S2). The observed enrichment pattern remained highly stable and persisted through week 12, maintaining a strong effect size and statistical significance. On the other hand, core structural pathways dedicated to methanogenesis, i.e., including methanol to methane (M00356) and acetate to methane (M00357), as well as one-carbon formaldehyde assimilation pathways (M00344, M00345, M00346) remained stationary near the centre line across during both sampling periods.

## Discussion

### Interaction between 3-nitrooxypropanol and direct-fed microbials supplementation

There was no significant overall interaction between 3-NOP and DFM supplementation in the current study. However, when data was analyzed on a weekly basis, negative interactions for CH_4_ intensity were noted from weeks 3 to 10. These interactions were likely due to the reduction effect of DFM supplementation on ECM led by the reduction in DMI. In contrast, 3-NOP significantly positively interacted (or tended to) with DFM supplementation in H_2_ intensity in weeks 4 and 5 and in weeks 3, 6, and 9, respectively. The reduction in CH_4_ emission caused by 3-NOP supplementation typically leads to increased H_2_ emission, as not all H_2_ redirected from the methanogenesis pathway can be utilized in alternative hydrogen sinks [10, 31]. The inclusion of *P. acidipropionici* strain P169 was expected to utilize excess H_2_ redirected from methanogenesis by 3-NOP administration as previous studies have shown that *P. acidipropionici* strain P169 promotes propionate formation [32, 33] utilizing hydrogen during this process [34]. 3-NOP supplementation led to increased abundance of certain genera such as *Prevotella*, and *Selenomonas. Prevotella* has been associated with healthy rumen and lower CH_4_ emissions in ruminants [35]. *Selenomonas ruminantium* has been stated as one of the succinate utilizer bacteria in the rumen, in which they reduce the dicarboxylic acids e.g., fumarate, malate and aspartate or their metabolites to propionate [4]. Therefore, the additional supplementation of *P. acidipropionici* strain P169 may have only had a limited or no impact on H_2_ consumption and propionate production [36]. One limitation of the present study was the absence of ruminal fluid samples, which precluded the assessment of ruminal fatty acid profiles across the experimental groups. This was unfortunate, since confirming a lack of evident enrichment in pathway modules associated with propionate production would further demonstrate that supplementations with *P. acidipropionici* did not promote propionate formation. Thus, further research is needed to explore the interactions between 3-NOP and DFMs on rumen fermentation profile.

### Effects of low dose 3-nitrooxypropanol supplementation

A recent meta-regression study suggested a 0.80 kg/d reduction in DMI with an average 3-NOP inclusion level of 77.0 mg/kg DMI, and DMI tended to be reduced 0.013 kg/d more with each unit increase in 3-NOP from its mean [37]. The dose of 3-NOP applied in the current study was far below 77.0 mg/kg and no significant effect of 3-NOP on DMI was observed. Aligned with the absent effect of 3-NOP on DMI, no effect was observed on milk yield and milk composition in the current study. Martins et al. [37] concluded that 3-NOP supplementation at levels much higher than which used in the current trial reduced milk yield by 0.98 kg/d, primarily due to the reduction effect in DMI.

In the meta-analysis by Kebreab et al. [3], the mitigation effect of 3-NOP on CH_4_ yield ranged from 16% to 60% across different dairy cattle studies which applied much higher dose of 3-NOP (37–132 mg/kg DM) than the dose implemented in the current study. In the current study, 3-NOP at the dose of 6.0 mg/kg DM only reduced CH_4_ emissions by 8% (production), 4% (yield), and 9% (intensity). In addition, the actual reductions observed in this study were much lower than the predicted reduction potentials based on diet composition according to the equations from Kebreab et al. [3]. The predicted reduction at the current dose and diet composition were 15%, 14%, and 16%, for production, yield and intensity, respectively. This indicates that the dose–response relationship of 3-NOP for methane mitigation may not be strictly linear at low doses, contrary to the prediction by Kebreab et al. [3], whose model was not tested at such low levels.

In the current experiment, cows receiving 3-NOP alone had higher *Megasphaera* abundance than CON animals. *Megasphaera elsdenii* is among the predominant lactate utilizing bacteria in the rumen. Recent studies have shown addition of *M. elsdenii* resulted in higher propionate production in lactating dairy cows [38]. Others have found higher abundance of genera such as *Succinivibrionaceae*, *Succiniclasticum*, and *Veillonellaceae* in animals fed with 3-NOP [39]. Martinez-Fernandez et al. [40] observed shift in rumen microbiome in 3-NOP supplemented cows with increases in *Succiniclasticum *spp. and *Candidatus saccharimonas* abundance. The genus *Succiniclasticum* is mainly propionate producing bacteria via the succinate pathway. These bacteria along with the *Succinivibrionaceae* have been linked with rapid hexose fermentation, particularly during higher dissolved hydrogen concentrations [39]. Tong et al. [41] revealed a positive correlation between ruminal *Candidatus saccharimonas* abundance and propionate production in the rumen of lactating dairy cows. In the current study, higher abundance of such bacteria was not detected. One plausible reason for such contradictory results could be the lower dosing rate of 3-NOP. Despite the lower dose of 3-NOP applied in this study, some bacteria species associated with propionate synthesis were still more abundant. The core methanogenesis pathway modules maintained a stable gene abundance centre line, which corroborates with earlier investigations on 3-NOP effect on reducing CH_4_ without any changing in the underlying the ruminal archaeal community [42]. Nonetheless, recent metagenomic studies showcased a shift in archaeal communities at species level upon supplementing 3-NOP in both dairy and beef cattle [43]. Such shift was not observed in the current experiment, however it is noteworthy that the 3-NOP dosing was lower than the effective dose use in previous experiments. Besides, highly significant enrichment of the non-oxidative Pentose Phosphate Pathway was observed upon 3-NOP supplementation particularly in later weeks of the experiment. This pathway provides ribose-5-phosphate for microbial nucleotide biosynthesis and serves as a major alternative pipeline for re-routing carbon structures. The enrichment of this pathway could imply that the non-methanogenic microbial community adapts by shifting excess carbon flows and reducing equivalents into carbohydrate shunts, consequently leading towards alternative biomass accumulation and cellular regeneration than enteric CH_4_.

### Effects of direct-fed microbials supplementation

DFM supplementation consistently reduced DMI across all experimental weeks in the current study. The effects of *B. licheniformis, B. subtilis*, and *P. acidipropionici* on DMI have been variable across studies. It has been suggested that *Bacillus* spp. is capable of producing a diverse range of enzymes, including fibrolytic, amylolytic, proteolytic, and lipolytic enzymes, which play important roles in nutrient digestion [44–46]. Therefore, a greater OM digestibility and subsequently a greater DMI were expected [47]. However, Francisco et al. [48] reported that feeding propionibacteria reduced DMI per kg of BW. In disagreement with our findings, Lamontagne et al. [49] and Oyebade et al. [50] reported an unaffected DMI, when a mixture of *B. licheniformis* and *B. subtilis* was included in the diet. Overall, the effects of DFM are inconsistent or even contradictory, as it depends on not only strains, species, and combination of DFM, but also dosage, diets, and animal related factors [5].

In this study, the effects of DFM supplementation on milk yield, ECM yield, and milk components varies across experimental weeks. The reduction in milk component yields was primarily attributed to the suppressed milk yield that resulted from the reduction in DMI. In contrast, Stein et al. [51] reported that *Propionibacterium* strain P169 supplementation tended to increase 4% FCM yield during weeks 1–12, and significantly increased it during weeks 13–25 at both high (6 × 10^11^ CFU/d) and low (6 × 10^10^ CFU/d) doses. However, Francisco et al. [48] observed no effect on 4% FCM yield over a 12-week period using a similar dosage of *Propionibacterium* strain P169. This discrepancy may be attributed to the shorter administration period in Francisco et al. [48], which may not have been sufficient to observe persistent lactation effects. These findings highlight the need for longer-term studies to further evaluate the effects of *Propionibacterium* supplementation on milk production. The effect of *Bacillus*-based DFMs on milk yield and composition were also variable in previous studies. Sun et al. [52] demonstrated increases in milk yield and milk constituents (fat, protein, and lactose), whereas, absence of effect of *Bacillus*-based DFMs on milk yield and composition and rumen microbiota and rumen fermentation parameters were reported in other studies [50, 53]. This difference on the effect of *Bacillus*-based DFMs on milk yield and composition might relate to the dose of DFMs applied, where Sun et al. [52] used a higher dose of *Bacillus subtilis* than our study. However, the stable or even increased milk yield during weeks 9 to 12 resulted in an improved feed efficiency (Fig. 1), as DMI remained unaffected. The interaction between DFM supplementation and experimental week demonstrated that the DFM supplementation reduced feed efficiency by 0.68% at week 3 and this negative effect gradually diminished with time and ended up with an increase in feed efficiency by 6.25% at week 12. The study by Terré et al. [53] did not observe significant effect on ECM per kg of DMI but reported that feeding a combination of *B. licheniformis* and *B. subtilis* improved kg milk/kg DMI by 3.2%. This difference might partly be related to the length of the experiment as the effect of DFM on the lactation performance seems to enhance along the time, and may be driven by the need for the rumen microbiome to adapt to the daily supplementation with a DFM. The study by Terré et al. [53] lasted 16 weeks including one adaptation week, whereas the experimental period in the present study was 3 weeks shorter, therefore, a more moderate effect should be expected. In addition, in this study, the DFM was a combination of *Propionibacterium acidipropionici* strain P169, *Bacillus subtilis*, and *B. licheniformis*, but only the two latter organisms in combination were used by Terré et al. [53], therefore, the results should be interpreted with caution.

Several DFMs have demonstrated CH_4_ reduction capabilities [54, 55]. However, the CH_4_ mitigation effects of DFM in previous studies have been inconsistent or even contradictory, which might be caused by the discrepancy among not only strains, species, and combinations of DFM, but also dosages, diets, feeding patterns and animal factors, such as age, physiological stage, health, and stress status [5]. In addition, viability and mode of action should also be considered when selecting DFMs. Although DFM supplementation hardly altered the microbiome diversity, the differential abundance of several species evidently changed in cows fed with the DFM mixture. Metagenomic analysis showed the abundance of both *Bacillus* and *Propionibacterium* was higher upon the addition of DFM, however the abundance of both *Bacillus* species declined at later stage of the experiment (Fig. S3). *Bacillus licheniformis* ferments glucose anaerobically, producing lactate and succinate, which can be converted to propionate via the acrylate and succinate pathways [56]. Vyas et al. [6] found that *Propionibacterium acidipropionici* strain P169 and P5, as well as *Propionibacterium jensenii* strain P54, reduced CH_4_ intensity by 12%, 8%, and 13%, respectively, in beef steers fed a high-forage diets, but had no effect on CH_4_ emission in beef steers consuming a high-grain diet [36]. In contrast, Wang et al. [55] reported that inclusion of *B. licheniformis* in the diet led to a reduction in CH_4_ production, whereas *B. subtilis* inclusion increased CH_4_ production*.* In the current study, different species within the *Lactobacillus* family had a higher abundance upon DFM supplementation. *Lactobacillus* are among the main lactic acid bacteria, in which has been proposed to reduce CH_4_ emission either through changes in rumen fermentation, direct rumen methanogenesis inhibition, or disrupting activity of certain ruminal bacteria that produce substrates for methanogenesis [57]. Recent in vivo works highlighted the potential effect of lactic acid bacteria strain type or the basal experimental diet on their methane mitigating outcome [54, 58, 59]. Nevertheless, as noted earlier, multiple factors can influence the CH₄ mitigation effect of DFMs and limited in vivo research exist on which one of the aforementioned factors has a more profound effect on the CH_4_ production. Hence, further research is needed to determine why no reduction was observed in this study. The bacteria-based supplementation introduced enrichment of the core biotin biosynthesis pathways, i.e., the classical prokaryotic pipeline via pimeloyl-ACP and alternative BioW pathway, throughout the current experiment. Biotin is a crucial metabolic cofactor required by both rumen microbes and the host animal for vital carboxylase enzyme functionalities, playing central roles in gluconeogenesis, fatty acid synthesis, and amino acid metabolism [60]. A meta-analysis by Chen et al. [61] indicated the use of biotin supplementation on increasing feed intake and milk production in lactating dairy cows. The simultaneous detection of both biotin pathways could suggest that DFM supplementation successfully enriched multiple distinct bacterial groups or taxa capable of diverse biotin production pathways.

## Conclusion

No significant interactions were observed between supplementation with an unexpectedly low dose of 3-NOP and DFM. Supplementation with 3-NOP at an unintended low level (6.0 mg/kg DM) altered the rumen bacterial community, reduced CH_4_ production and intensity, and led to H_2_ accumulation, without affecting DMI or milk yield. However, the reductions in CH_4_ yield and intensity were less than anticipated, suggesting a non-linear relationship between dosage and response. DFM supplementation resulted in decreased DMI but did not influence ECM yield, thereby improving feed efficiency. Notably, increased feed efficiency was only observed at the conclusion of the trial, indicating that a microbial adaptation period may be required. Although DFM altered microbiome composition, these changes did not lead to reduced CH₄ emission or enhanced production performance when administered in combination with 3-NOP.

## Supplementary Information

Additional file 1: Table S1. Effects of 3-NOP and direct-fed microbials (DFM) on milk and energy-corrected milk yield over time. Table S2. Effects of 3-NOP and direct-fed microbials (DFM) on feed efficiencyover time. Table S3. Effects of 3-NOP and direct-fed microbials (DFM) on CO_2_ production, yield, and intensity over time. Table S4. Effects of 3-NOP and direct-fed microbials (DFM) on CH_4_ production, yield, and intensity over time. Table S5. Effects of 3-NOP and direct-fed microbials (DFM) on H_2_ production, yield, and intensity over time.

Additional file 2: Fig. S1. Volcano plots displaying differential abundance of KEGG pathway modules between Control and NOP groups. Fig. S2. Volcano plots displaying differential abundance of KEGG pathway modules between Control and DFM groups. Fig. S3. Relative abundance of *Bacillus *species in the oral-derived rumen bacterial community over time.

## Data Availability

All data supporting the findings of this study are available from the corresponding author upon reasonable request.

## Abbreviations

3-NOP: 3-Nitrooxypropanol
ADG: Average daily body weight gain
BW: Body weight
CH_4_: Methane
CO_2_: Carbon dioxide
CON: Control
CP: Crude protein
DFM: Direct-fed microbials
DIM: Days in milk
DMI: Dry matter intake
ECM: Energy-corrected milk
H_2_: Hydrogen
NDF: Neutral detergent fibre
NOP: Basal PMR diet with 3-NOP supplementation
NOPDFM: Basal PMR diet with combination of 3-NOP and mixture of *P. acidipropionici* strain P169, *B. subtilis*, and *B. licheniformis* supplementation
PMR: Partial mixed ration
