# Supplementary material for: Effects of a low dose of 3-nitrooxypropanol and a combination of direct-fed microbials on methane emission, oral swab bacterial community, and performance in dairy cows
Source: J Anim Sci Biotechnol. 2026 Oct 7;17:189. doi: 10.1186/s40104-026-01501-z (PMC13644272; doi:10.1186/s40104-026-01501-z)
Supplement: Supplementary file 1 [file 40104_2026_1501_MOESM1_ESM.docx]

**Table S1** Effects of 3-NOP and direct-fed microbials (DFM) on milk and energy-corrected milk yield over time^1^

| **Items^2^** | **Experimental week** | | | | | | | | | |
| --- | --- | --- | --- | --- | --- | --- | --- | --- | --- | --- |
|  | **3** | **4** | **5** | **6** | **7** | **8** | **9** | **10** | **11** | **12** |
| Milk |  |  |  |  |  |  |  |  |  |  |
| CON | 37.2 | 37.0 | 36.4 | 35.8 | 35.0 | 34.5 | 34.3 | 34.2 | 34.2 | 33.9 |
| NOP | 37.5 | 37.1 | 36.6 | 35.9 | 35.5 | 35.2 | 34.7 | 34.1 | 33.7 | 33.8 |
| DFM | 34.7 | 34.6 | 33.9 | 33.3 | 33.0 | 33.4 | 33.6 | 33.8 | 33.9 | 33.8 |
| NOPDFM | 35.0 | 34.9 | 34.2 | 33.4 | 32.6 | 32.1 | 32.2 | 32.6 | 33.1 | 33.9 |
| SEM^3^ | 1.58 | 1.59 | 1.56 | 1.52 | 1.42 | 1.34 | 1.33 | 1.35 | 1.38 | 1.40 |
| *P*-value^4^ |  |  |  |  |  |  |  |  |  |  |
| NOP | 0.52 | 0.82 | 0.77 | 0.85 | 0.95 | 0.72 | 0.54 | 0.46 | 0.51 | 0.99 |
| DFM | <0.001 | <0.01 | <0.01 | <0.01 | <0.01 | 0.01 | 0.06 | 0.29 | 0.63 | 0.99 |
| NOP × DFM | 0.99 | 0.91 | 0.96 | 0.99 | 0.63 | 0.25 | 0.30 | 0.58 | 0.88 | 0.92 |
| ECM |  |  |  |  |  |  |  |  |  |  |
| CON | 36.3 | 37.1 | 36.9 | 36.8 | 36.2 | 35.7 | 35.4 | 35.5 | 35.4 | 35.2 |
| NOP | 37.6 | 37.7 | 37.3 | 36.7 | 36.6 | 36.3 | 36.3 | 35.8 | 35.7 | 35.4 |
| DFM | 35.5 | 35.7 | 35.5 | 34.9 | 34.8 | 35.0 | 35.5 | 35.9 | 36.2 | 36.2 |
| NOPDFM | 36.1 | 36.3 | 36.0 | 35.3 | 34.8 | 34.4 | 34.8 | 35.5 | 36.3 | 36.2 |
| SEM^3^ | 1.33 | 1.32 | 1.32 | 1.31 | 1.26 | 1.18 | 1.20 | 1.25 | 1.27 | 1.28 |
| *P*-value^4^ |  |  |  |  |  |  |  |  |  |  |
| NOP | 0.10 | 0.32 | 0.51 | 0.77 | 0.83 | 0.97 | 0.92 | 0.94 | 0.84 | 0.90 |
| DFM | 0.04 | 0.02 | 0.04 | 0.02 | 0.03 | 0.10 | 0.44 | 0.93 | 0.49 | 0.37 |
| NOP × DFM | 0.55 | 0.98 | 0.99 | 0.70 | 0.76 | 0.42 | 0.37 | 0.72 | 0.89 | 0.90 |

^1^There were 12 observations for each treatment in each week

^2^CON: without any supplementation; NOP: 3-NOP; DFM: mixture of *Propionibacterium acidipropionici* strain P169, *Bacillus. subtilis*, and *B. licheniformis*; NOPDFM: combination of 3-NOP and mixture of *Propionibacterium acidipropionici* strain P169, *B. subtilis*, and *B. licheniformis*; ECM: energy-corrected milk

^3^Largest values were reported, due to missing observations

^4^The variable in the covariate period always had a significance of *P* < 0.001

**Table S2** Effects of 3-NOP and direct-fed microbials (DFM) on feed efficiency (ECM/DMI) over time^1^

| **Items^2^** | **Experimental week** | | | | | | | | | |
| --- | --- | --- | --- | --- | --- | --- | --- | --- | --- | --- |
|  | **3** | **4** | **5** | **6** | **7** | **8** | **9** | **10** | **11** | **12** |
| CON | 1.47 | 1.46 | 1.45 | 1.44 | 1.41 | 1.42 | 1.44 | 1.45 | 1.42 | 1.40 |
| NOP | 1.51 | 1.47 | 1.47 | 1.43 | 1.41 | 1.40 | 1.41 | 1.39 | 1.39 | 1.39 |
| DFM | 1.45 | 1.42 | 1.41 | 1.38 | 1.37 | 1.37 | 1.39 | 1.41 | 1.42 | 1.44 |
| NOPDFM | 1.49 | 1.48 | 1.46 | 1.43 | 1.41 | 1.39 | 1.43 | 1.44 | 1.46 | 1.48 |
| SEM^3^ | 0.03 | 0.03 | 0.04 | 0.04 | 0.04 | 0.04 | 0.03 | 0.04 | 0.04 | 0.04 |
| *P*-value^4^ |  |  |  |  |  |  |  |  |  |  |
| NOP | 0.08 | 0.17 | 0.23 | 0.42 | 0.55 | 0.86 | 0.65 | 0.85 | 0.82 | 0.56 |
| DFM | 0.44 | 0.42 | 0.44 | 0.31 | 0.55 | 0.34 | 0.57 | 0.77 | 0.28 | 0.08 |
| NOP × DFM | 0.99 | 0.38 | 0.65 | 0.43 | 0.56 | 0.49 | 0.27 | 0.21 | 0.31 | 0.27 |

^1^Observations with peculiar feed intake records were omitted as follows: CON group: 1 observation each from weeks 3, 4, 5, 6, and 7; 2 observations each from weeks 8, 11, and 12; 3 observations each from weeks 9 and 10. NOP group: 1 observation each from weeks 5, 6, 7, 8, 9, and 10

^2^CON: without any supplementation; NOP: 3-NOP; DFM: mixture of *Propionibacterium acidipropionici* strain P169, *Bacillus. subtilis*, and *B. licheniformis*; NOPDFM: combination of 3-NOP and mixture of *Propionibacterium acidipropionici* strain P169, *B. subtilis*, and *B. licheniformis*

^3^Largest values were reported, due to missing observations

^4^The variable in the covariate period always had a significance of *P* < 0.001

**Table S3** Effects of 3-NOP and direct-fed microbials (DFM) on CO_2_ production, yield, and intensity over time^1^

| **Items^2^** | **Experimental week** | | | | | | | | | |
| --- | --- | --- | --- | --- | --- | --- | --- | --- | --- | --- |
|  | **3** | **4** | **5** | **6** | **7** | **8** | **9** | **10** | **11** | **12** |
| CO_2_ production, g/d | |  |  |  |  |  |  |  |  |  |
| CON | 13540 | 13570 | 13649 | 13792 | 13779 | 13918 | 14026 | 14296 | 14095 | 14141 |
| NOP | 13109 | 13088 | 13180 | 13477 | 13728 | 13949 | 14005 | 13949 | 13837 | 14019 |
| DFM | 13420 | 13696 | 13672 | 13873 | 13611 | 13856 | 13939 | 14161 | 14160 | 14002 |
| NOPDFM | 13330 | 13093 | 13313 | 13464 | 13519 | 13782 | 13910 | 14143 | 13874 | 14019 |
| SEM^3^ | 406 | 330 | 360 | 366 | 427 | 399 | 427 | 468 | 439 | 426 |
| *P*-value^4^ |  |  |  |  |  |  |  |  |  |  |
| NOP | 0.37 | 0.01 | 0.10 | 0.18 | 0.82 | 0.94 | 0.94 | 0.62 | 0.41 | 0.87 |
| DFM | 0.87 | 0.75 | 0.76 | 0.90 | 0.55 | 0.72 | 0.79 | 0.95 | 0.88 | 0.82 |
| NOP × DFM | 0.55 | 0.78 | 0.83 | 0.86 | 0.95 | 0.87 | 0.99 | 0.66 | 0.97 | 0.83 |
| CO_2_ yield, g/kg DMI | |  |  |  |  |  |  |  |  |  |
| CON | 550 | 537 | 533 | 533 | 530 | 538 | 554 | 566 | 550 | 554 |
| NOP | 530 | 524 | 533 | 539 | 548 | 544 | 546 | 544 | 534 | 540 |
| DFM | 543 | 539 | 535 | 539 | 527 | 530 | 531 | 540 | 545 | 544 |
| NOPDFM | 554 | 544 | 549 | 553 | 556 | 564 | 573 | 578 | 563 | 564 |
| SEM^3^ | 14.0 | 13.7 | 11.5 | 12.4 | 15.3 | 14.0 | 14.8 | 16.3 | 16.3 | 16.0 |
| *P*-value^4^ |  |  |  |  |  |  |  |  |  |  |
| NOP | 0.78 | 0.76 | 0.45 | 0.39 | 0.05 | 0.08 | 0.14 | 0.50 | 0.87 | 0.79 |
| DFM | 0.51 | 0.38 | 0.40 | 0.40 | 0.82 | 0.66 | 0.86 | 0.73 | 0.35 | 0.59 |
| NOP × DFM | 0.23 | 0.47 | 0.51 | 0.70 | 0.64 | 0.28 | 0.06 | 0.05 | 0.21 | 0.24 |
| CO_2_ intensity, g/kg ECM | | |  |  |  |  |  |  |  |  |
| CON | 386 | 381 | 385 | 392 | 398 | 407 | 413 | 422 | 417 | 423 |
| NOP | 351 | 354 | 356 | 369 | 378 | 384 | 387 | 390 | 385 | 389 |
| DFM | 375 | 381 | 379 | 390 | 386 | 388 | 385 | 383 | 385 | 380 |
| NOPDFM | 375 | 370 | 381 | 393 | 402 | 411 | 411 | 413 | 393 | 385 |
| SEM^3^ | 12.2 | 11.8 | 11.1 | 12.0 | 13.6 | 13.3 | 13.9 | 15.1 | 15.2 | 15.3 |
| *P*-value^4^ |  |  |  |  |  |  |  |  |  |  |
| NOP | 0.13 | 0.05 | 0.18 | 0.40 | 0.94 | 0.92 | 0.98 | 0.93 | 0.43 | 0.34 |
| DFM | 0.58 | 0.43 | 0.35 | 0.30 | 0.70 | 0.77 | 0.82 | 0.54 | 0.42 | 0.10 |
| NOP × DFM | 0.10 | 0.39 | 0.11 | 0.24 | 0.13 | 0.07 | 0.05 | 0.03 | 0.16 | 0.18 |

^1^For CO_2_ yield, observations with peculiar feed intake records were omitted as follows: CON group: 1 observation each from weeks 3, 4, 5, 6, and 7; 2 observations each from weeks 8, 11, and 12; 3 observations each from weeks 9 and 10. NOP group: 1 observation each from weeks 5, 6, 7, 8, 9, and 10. Otherwise, there were 12 observations for each treatment in each week

^2^CON: without any supplementation; NOP: 3-NOP; DFM: mixture of *Propionibacterium acidipropionici* strain P169, *Bacillus. subtilis*, and *B. licheniformis*; NOPDFM: combination of 3-NOP and mixture of *Propionibacterium acidipropionici* strain P169, *B. subtilis*, and *B. licheniformis*

^3^Largest values were reported, due to missing observations

^4^The variable in the covariate period always had a significance of *P* < 0.001.

**Table S4** Effect of 3-NOP and direct-fed microbials (DFM) on CH_4_ production, yield, and intensity over time^1^

| **Items^2^** | **Experimental week** | | | | | | | | | |
| --- | --- | --- | --- | --- | --- | --- | --- | --- | --- | --- |
|  | **3** | **4** | **5** | **6** | **7** | **8** | **9** | **10** | **11** | **12** |
| CH_4_ production, g/d | |  |  |  |  |  |  |  |  |  |
| CON | 366 | 372 | 373 | 369 | 365 | 366 | 373 | 384 | 380 | 382 |
| NOP | 323 | 316 | 314 | 317 | 326 | 334 | 342 | 340 | 343 | 348 |
| DFM | 349 | 360 | 359 | 358 | 346 | 351 | 359 | 363 | 376 | 369 |
| NOPDFM | 330 | 327 | 333 | 333 | 337 | 343 | 352 | 356 | 348 | 357 |
| SEM^3^ | 12.8 | 11.4 | 10.7 | 11.2 | 14.6 | 13.5 | 14.1 | 15.7 | 14.8 | 15.9 |
| *P*-value^4^ |  |  |  |  |  |  |  |  |  |  |
| NOP | <0.01 | <0.001 | <0.001 | <0.001 | 0.08 | 0.12 | 0.17 | 0.08 | 0.01 | 0.12 |
| DFM | 0.60 | 0.86 | 0.82 | 0.82 | 0.73 | 0.82 | 0.86 | 0.83 | 0.98 | 0.85 |
| NOP × DFM | 0.29 | 0.23 | 0.09 | 0.22 | 0.28 | 0.34 | 0.38 | 0.20 | 0.73 | 0.44 |
| CH_4_ yield, g/kg DM | |  |  |  |  |  |  |  |  |  |
| CON | 15.0 | 14.8^a^ | 14.6^a^ | 14.4 | 14.2 | 14.3 | 14.8 | 15.2 | 14.7 | 14.7 |
| NOP | 13.0 | 12.6^b^ | 12.8^b^ | 12.8 | 13.1 | 13.1 | 13.4 | 13.3 | 13.4 | 13.5 |
| DFM | 14.0 | 14.1^ab^ | 14.0^ab^ | 13.9 | 13.3 | 13.4 | 13.6 | 13.8 | 14.5 | 14.3 |
| NOPDFM | 13.8 | 13.7^ab^ | 13.9^ab^ | 13.8 | 14.0 | 14.1 | 14.6 | 14.7 | 14.2 | 14.5 |
| SEM^3^ | 0.49 | 0.47 | 0.40 | 0.45 | 0.59 | 0.52 | 0.53 | 0.61 | 0.60 | 0.65 |
| *P*-value^4^ |  |  |  |  |  |  |  |  |  |  |
| NOP | 0.03 | <0.01 | 0.01 | 0.06 | 0.81 | 0.77 | 0.86 | 0.47 | 0.12 | 0.42 |
| DFM | 0.95 | 0.77 | 0.60 | 0.56 | 0.97 | 0.93 | 0.99 | 0.92 | 0.57 | 0.63 |
| NOP × DFM | 0.06 | 0.04 | 0.02 | 0.07 | 0.11 | 0.04 | 0.01 | 0.02 | 0.32 | 0.23 |
| CH_4_ intensity, g/kg ECM | | |  |  |  |  |  |  |  |  |
| CON | 10.5^a^ | 10.4^a^ | 10.5^a^ | 10.5^a^ | 10.5 | 10.7 | 11.0 | 11.4^a^ | 11.3 | 11.4 |
| NOP | 8.47^b^ | 8.42^b^ | 8.47^b^ | 8.63^b^ | 8.97 | 9.10 | 9.38 | 9.43^b^ | 9.46 | 9.62 |
| DFM | 9.73^ab^ | 9.98^a^ | 9.97^a^ | 10.1^a^ | 9.82 | 9.88 | 9.96 | 9.92^ab^ | 10.3 | 10.07 |
| NOPDFM | 9.35^ab^ | 9.33^ab^ | 9.55^ab^ | 9.73^ab^ | 10.0 | 10.3 | 10.4 | 10.3^ab^ | 9.83 | 9.81 |
| SEM^3^ | 0.39 | 0.38 | 0.34 | 0.38 | 0.46 | 0.43 | 0.43 | 0.48 | 0.48 | 0.55 |
| *P*-value^4^ |  |  |  |  |  |  |  |  |  |  |
| NOP | <0.01 | <0.001 | <0.001 | <0.01 | 0.15 | 0.17 | 0.20 | 0.12 | 0.02 | 0.06 |
| DFM | 0.91 | 0.62 | 0.39 | 0.33 | 0.76 | 0.73 | 0.93 | 0.53 | 0.49 | 0.23 |
| NOP × DFM | 0.03 | 0.04 | <0.01 | 0.04 | 0.04 | 0.02 | 0.02 | 0.01 | 0.15 | 0.14 |

^a,b^ LS-means to differ between treatments within column, if there is significant interaction

^1^For CH_4_ yield, observations with peculiar feed intake records were omitted as follows: CON group: 1 observation each from weeks 3, 4, 5, 6, and 7; 2 observations each from weeks 8, 11, and 12; 3 observations each from weeks 9 and 10. NOP group: 1 observation each from weeks 5, 6, 7, 8, 9, and 10. Otherwise, there were 12 observations for each treatment in each week

^2^CON: without any supplementation; NOP: 3-NOP; DFM: mixture of *Propionibacterium acidipropionici* strain P169, *Bacillus. subtilis*, and *B. licheniformis*; NOPDFM: combination of 3-NOP and mixture of *Propionibacterium acidipropionici* strain P169, *B. subtilis*, and *B. licheniformis*

^3^Largest values were reported, due to missing observations

^4^The variable in the covariate period always had a significance of *P* ≤ 0.05

**Table S5** Effect of 3-NOP and direct-fed microbials (DFM) on H_2_ production, yield, and intensity over time^1^

| **Items^2^** | **Experimental week** | | | | | | | | | |
| --- | --- | --- | --- | --- | --- | --- | --- | --- | --- | --- |
|  | **3** | **4** | **5** | **6** | **7** | **8** | **9** | **10** | **11** | **12** |
| H_2_ production, g/d | |  |  |  |  |  |  |  |  |  |
| CON | 2.54 | 2.62 | 2.70 | 2.65 | 2.69 | 2.68 | 2.77 | 2.83 | 2.82 | 2.94 |
| NOP | 4.13 | 4.02 | 4.02 | 4.10 | 4.03 | 4.18 | 4.00 | 3.95 | 4.08 | 4.27 |
| DFM | 2.53 | 2.71 | 2.69 | 2.72 | 2.61 | 2.75 | 2.85 | 2.91 | 2.98 | 2.79 |
| NOPDFM | 4.52 | 4.68 | 4.79 | 4.78 | 4.58 | 4.59 | 4.65 | 4.70 | 4.72 | 4.93 |
| SEM^3^ | 0.26 | 0.27 | 0.33 | 0.33 | 0.32 | 0.32 | 0.31 | 0.36 | 0.38 | 0.39 |
| *P*-value^4^ |  |  |  |  |  |  |  |  |  |  |
| NOP | <0.001 | <0.001 | <0.001 | <0.001 | <0.001 | <0.001 | <0.001 | <0.001 | <0.001 | <0.001 |
| DFM | 0.33 | 0.10 | 0.15 | 0.18 | 0.39 | 0.38 | 0.19 | 0.22 | 0.23 | 0.51 |
| NOP × DFM | 0.29 | 0.18 | 0.13 | 0.26 | 0.21 | 0.51 | 0.28 | 0.32 | 0.47 | 0.27 |
| H_2_ yield, g/kg DMI | |  |  |  |  |  |  |  |  |  |
| CON | 0.102 | 0.102 | 0.104 | 0.102 | 0.104 | 0.103 | 0.106 | 0.109 | 0.106 | 0.113 |
| NOP | 0.164 | 0.160 | 0.158 | 0.157 | 0.151 | 0.159 | 0.154 | 0.153 | 0.156 | 0.163 |
| DFM | 0.101 | 0.105 | 0.104 | 0.105 | 0.100 | 0.104 | 0.107 | 0.111 | 0.116 | 0.110 |
| NOPDFM | 0.184 | 0.192 | 0.196 | 0.195 | 0.188 | 0.187 | 0.191 | 0.191 | 0.190 | 0.197 |
| SEM^3^ | 0.008 | 0.009 | 0.011 | 0.012 | 0.011 | 0.012 | 0.012 | 0.014 | 0.014 | 0.015 |
| *P*-value^4^ |  |  |  |  |  |  |  |  |  |  |
| NOP | <0.001 | <0.001 | <0.001 | <0.001 | <0.001 | <0.001 | <0.001 | <0.001 | <0.001 | <0.001 |
| DFM | 0.15 | 0.05 | 0.06 | 0.06 | 0.12 | 0.15 | 0.07 | 0.13 | 0.07 | 0.29 |
| NOP × DFM | 0.13 | 0.09 | 0.04 | 0.08 | 0.04 | 0.18 | 0.09 | 0.17 | 0.32 | 0.21 |
| H_2_ intensity, g/kg ECM | |  |  |  |  |  |  |  |  |  |
| CON | 0.071 | 0.072^c^ | 0.075^c^ | 0.074 | 0.076 | 0.077 | 0.079 | 0.082 | 0.083 | 0.088 |
| NOP | 0.107 | 0.104^b^ | 0.104^b^ | 0.108 | 0.110 | 0.112 | 0.110 | 0.109 | 0.112 | 0.117 |
| DFM | 0.071 | 0.076^c^ | 0.075^c^ | 0.077 | 0.075 | 0.078 | 0.079 | 0.080 | 0.083 | 0.077 |
| NOPDFM | 0.125 | 0.130^a^ | 0.134^a^ | 0.136 | 0.134 | 0.137 | 0.137 | 0.136 | 0.132 | 0.133 |
| SEM^3^ | 0.005 | 0.006 | 0.007 | 0.008 | 0.008 | 0.008 | 0.008 | 0.010 | 0.010 | 0.010 |
| *P*-value^4^ |  |  |  |  |  |  |  |  |  |  |
| NOP | <0.001 | <0.001 | <0.001 | <0.001 | <0.001 | <0.001 | <0.001 | <0.001 | <0.001 | <0.001 |
| DFM | 0.06 | <0.01 | 0.02 | 0.04 | 0.15 | 0.11 | 0.10 | 0.21 | 0.28 | 0.84 |
| NOP × DFM | 0.07 | 0.03 | 0.02 | 0.09 | 0.11 | 0.14 | 0.09 | 0.13 | 0.29 | 0.18 |

^a,b^LS-means to differ between treatments within column, if there is significant interaction

^1^For H_2_ yield, observations with peculiar feed intake records were omitted as follows: CON group: 1 observation each from weeks 3, 4, 5, 6, and 7; 2 observations each from weeks 8, 11, and 12; 3 observations each from weeks 9 and 10. NOP group: 1 observation each from weeks 5, 6, 7, 8, 9, and 10. Otherwise, there were 12 observations for each treatment in each week

^2^CON: without any supplementation; NOP: 3-NOP; DFM: mixture of *Propionibacterium acidipropionici* strain P169, *Bacillus. subtilis*, and *B. licheniformis*; NOPDFM: combination of 3-NOP and mixture of *Propionibacterium acidipropionici* strain P169, *B. subtilis*, and *B. licheniformis*

^3^Largest values were reported, due to missing observations

^4^The variable in the covariate period always had a significance of *P* < 0.05
