# Supplementary material for: Effects of a low dose of 3-nitrooxypropanol and a combination of direct-fed microbials on methane emission, oral swab bacterial community, and performance in dairy cows
Source: J Anim Sci Biotechnol. 2026 Oct 7;17:189. doi: 10.1186/s40104-026-01501-z (PMC13644272; doi:10.1186/s40104-026-01501-z)
Supplement: Supplementary file 2 [file 40104_2026_1501_MOESM2_ESM.docx]

**Fig. S1** Volcano plots displaying differential abundance of KEGG pathway modules between Control and NOP groups. **A** Functional shifts at week6 depicting non-significant shifts. **B** Functional profiles at week12 displaying the highly significant enrichment of the Pentose Phosphate Pathway (M00004, orange dot) under NOP treatment. Vertical dashed lines denote biological effect size limits (± 0.50). The horizontal dashed line defines the strict unadjusted statistical significance threshold. Grey dots point to modules with negligible variance, whereas blue and orange dots highlight localized functional differences

**Fig. S2** Volcano plots displaying differential abundance of KEGG pathway modules between Control and DFM groups. **A** Functional shifts at wk6 highlighting early significant enrichment of biotin biosynthesis modules (M00123 and M00577, orange dots). **B** Functional landscape at week12 demonstrating sustained biotin module enrichment (M00123 and M00577, orange dots). Vertical dashed lines denote biological effect size limits (±0.50). The horizontal dashed line defines the strict unadjusted statistical significance threshold. Grey dots point to modules with negligible variance, while blue and orange dots highlight localized functional differences

**Fig. S3** Relative abundance of *Bacillus* species in the oral-derived rumen bacterial community over time
